# Understanding grain development in the Poaceae family by comparing conserved and distinctive pathways through omics studies in wheat and maize

**DOI:** 10.3389/fpls.2024.1393140

**Published:** 2024-07-18

**Authors:** Yuanyuan Ji, Thulani Hewavithana, Andrew G. Sharpe, Lingling Jin

**Affiliations:** ^1^ Department of Computer Science, University of Saskatchewan, Saskatoon, SK, Canada; ^2^ Global Institute for Food Security, University of Saskatchewan, Saskatoon, SK, Canada

**Keywords:** seed development, omics, C3 plant, C4 plant, wheat, maize, genomics, transcriptome

## Abstract

The Poaceae family, commonly known as the grass family, encompasses a diverse group of crops that play an essential role in providing food, fodder, biofuels, environmental conservation, and cultural value for both human and environmental well-being. Crops in Poaceae family are deeply intertwined with human societies, economies, and ecosystems, making it one of the most significant plant families in the world. As the major reservoirs of essential nutrients, seed grain of these crops has garnered substantial attention from researchers. Understanding the molecular and genetic processes that controls seed formation, development and maturation can provide insights for improving crop yield, nutritional quality, and stress tolerance. The diversity in photosynthetic pathways between C3 and C4 plants introduces intriguing variations in their physiological and biochemical processes, potentially affecting seed development. In this review, we explore recent studies performed with omics technologies, such as genomics, transcriptomics, proteomics and metabolomics that shed light on the mechanisms underlying seed development in wheat and maize, as representatives of C3 and C4 plants respectively, providing insights into their unique adaptations and strategies for reproductive success.

## 1 Introduction

Plant seeds are remarkable vessels of life, encapsulating the potential for growth and ensuring the survival and dispersal of the species. They originate from the maturation of ovules within flowering plant, containing an embryo surrounded by a protective outer layer called the seed coat. The genetic repository within the embryo controls the entire life cycle of the plant and the seed coat shields it from environmental stresses and pathogens securing the unfolding of the plant’s next generation in interaction with the environment (Kigel, 1995). Seeds stores the vital components of nutrient that nourish the development of a plant until it can photosynthesize on its own. As the seedling germinates, the embryo and cotyledons surrounded by the endosperm start to grow. Endosperm is a nutrient-rich tissue that mainly contains starch and provides essential energy to the germinating seedling. This resource allocation mechanism diverges between monocots and dicots. Monocots, characterized by a single cotyledon which is an embryonic leaf in the germinating seed, tend to retain their endosperm to nourish the growing seedling. Conversely, dicots often transfer nutrients from the endosperm to the cotyledons as the seed matures (Sabelli, 2012; Sreenivasulu and Wobus, 2013).

Approximately 60% of human energy supply is derived from three monocots cereal species — wheat, rice and maize, which all belong to the Poaceae family making Poaceae the most culturally and economically important plant family in the world (Linder et al., 2018). Therefore, seed development in the Poaceae family has been investigated through various approaches, including biochemistry, molecular biology and omics studies. There are studies describing the genotype/trait associations, genetics and transcriptional regulatory network of seeds development in wheat, rice and maize respectively (Dai et al., 2021; Shikha et al., 2021; Chen et al., 2022; Wang and Sun, 2023). However, no comparative analyses of omics data sets between wheat and maize in Poaceae family exist to the best of our knowledge. Therefore, understanding molecular and genetic mechanisms, modeling of biological networks by interrogation of entire pools of genomic, transcriptomic, metabolomic and proteomic data sets from the two crops with extensive comparative analysis remains a substantially important goal to further seed yield improvements.

Furthermore, it is well-established that the C4 pathway involves the incorporation of carbon into 4-carbon metabolites like malate and oxaloacetate, while the C3 photosynthetic pathway fixes *CO*_2_ into 3-carbon metabolites such as 3-phosphoglycerate (PGA) through the Calvin cycle. There has been an ongoing debate surrounding whether wheat, a representative C3 photosynthetic crop, utilizes the C4 photosynthetic pathway during grain development like maize, in recent decades (Hibberd and Furbank, 2016). Omics studies have the potential to provide insights on certain questions regarding to this topic.

In the past decade, high-throughput sequencing technologies have revolutionized entire branches of the life sciences including plant improvement, human disease, pharmaceutical engineering. The term ‘omics’ is derived from genomics and signifies a holistic approach to the study of biological systems. It involves the comprehensive investigation of entire set of biological molecules and processes on a large scale, providing an in-depth understanding of complex biological systems (Hasin et al., 2017). There are many types of omics, each focusing on a specific aspect of biological data. Several of them have widely applied in plant science as described below.

1. Genomics is a study of the complete genome of a particular species. It focuses on sequencing of an entire genome and identifying genetic variants associated with plant traits or responses. In the plant research field, genome-wide association studies (GWAS) and quantitative trait locus (QTL) analysis are the most popular approaches to discover variants of interest associated with various plant traits (Korte and Farlow, 2013).

2. Transcriptomics is a study of entire RNA molecules transcribed in a cell or organism. It is mainly used for analysis of gene expression levels, alternative splicing, and non-coding RNA. RNA-Seq, a widely used short-read sequencing technology, offers significantly more accurate transcriptome profiling, enabling the detection of specific isoforms with greater precision compared to other methodologies like microarrays (Wang et al., 2009).

3. Proteomics is a comprehensive analysis of the entire protein components in a specific tissue from a particular species. Utilizing mass spectrometry (MS)-based proteomics, it is possible to characterize and quantify thousands of proteins simultaneously, and uncover their post-translational modifications (PTMs) in a parallel manner (Bantscheff et al., 2012; Dupree et al., 2020). Presently, the insights gleaned from proteomic research has substantially enhanced our comprehension of biological complexity. This advancement has improved our understanding of the molecular mechanisms driving plant responses to environmental stimuli and various developmental stages.

4. Metabolomics focuses on the analysis of small molecules (metabolites) within a biological system (cell, tissue, organism, etc). It offers a comprehensive snapshot of a plant’s metabolic profile, encompassing the identification and quantification of diverse compounds like sugars, organic acids, and secondary metabolites. Notably, these plant secondary metabolites confer a multitude of advantages, including protective functions for the plants and health-promoting properties for consumers (Zhu et al., 2016; Kumar et al., 2017). By characterizing novel metabolites and examining their dynamic flows, we can significantly deepen our understanding of the pathways through which plants synthesize and regulate these vital compounds. These compounds are crucial not only for plant growth and development but also for their ability to respond effectively to various stressors.

Additionally, a range of omics disciplines, such as epigenomics, phenomics, and lipidomics, play a pivotal role in advancing research on plant seed development. This review is dedicated to offering a thorough overview of the current understanding of the molecular facets of wheat and maize seed development. Specifically, it focuses on delineating both the commonalities and differences between these two vital crops within the context of the aforementioned four omics domains.

## 2 Seed development in wheat and maize

**Figure 1:**
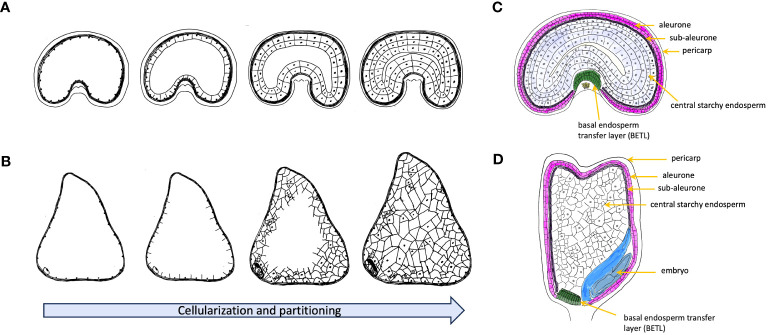
Schematic representation of early endosperm cellularization process before accumulation of storage metabolites for wheat **(A)** and maize **(B)**. Nuclear division initially in the coenocyte forms a multinucleate structure and then moves towards to the peripheral part of the multinucleate coenocyte. With formation of the first anticlinal cell walls, nuclear divisions shift towards to the center of multinucleate endosperm. The process continues with further growth moving inwards until cellularization is completed. Wheat embryo develops a uniform partitioning endosperm, and maize forms an irregular distribution of the cell division. **(C)** Schematic representation of cross section of a developing grain of wheat. **(D)** Schematic representation of longitudinal section of a developing grain of maize.

The development of seeds is a complex process encompassing several maternal and filial tissues with a series of intricate events. These events begin with the fertilization of the ovule where one sperm cell fuses with the egg cell to form a zygote marking the start of the embryonic development. The cellularization events followed after fertilization and lead to the formation of triploid endosperm (two polar nuclei and one sperm cell) with continuous cell division (Kowles and Phillips, 1988; Berger, 1999). Unlike dicot species, that are dominated by the developing embryo, the endosperm in cereals like wheat and maize will continuously accumulates starch and storage proteins leading to a high-calories content. While the endosperms of cereal grains, like wheat, generally achieve full cellularization through ongoing cell division and alveolation (**Figure 1**), the endosperm of maize displays a somewhat distinct pattern of cellularization. Contrasting with wheat, where maize individual cells form distinct cell walls, the primary endosperm cell undergoes several rounds of nuclear divisions without the formation of cell walls. As a result, the endosperm remains multinucleate, with many nuclei sharing a common cytoplasm (Olsen et al., 1995). Hence, maize demonstrates an irregular final partitioning of the central vacuole at the base of its endosperm, which significantly influences the kernel size and contributes to its distinctive morphology (Leroux et al., 2014) (**Figure 1**). At the end of mitotic division, the development of the outer and inner layers, known as the pericarp and aleurone layer, respectively, signals the commencement of the grain filling stage. Owing to the reduced mitotic activity and limited cell size, the growth of the endosperm is predominantly driven by cell enlargement, characterized by the accumulation of starch, lipids, and proteins (Randolph, 1936; Kowles and Phillips, 1985; Li et al., 2006; Kong et al., 2015). Studies on the distribution and retention of radioactivity of ^14^ *CO*_2_ on cereal grains suggested that photosynthesis plays a crucial role in sustaining the majority of the grain weight (Merah and Monneveux, 2015; Yang et al., 2016; Estévez-Geffriaud et al., 2020). However, the sources of reserves remobilized for seed grain development vary between wheat and maize. In maize, although stored carbohydrates are available for seed growth, they are primarily allocated to maintenance processes, especially under significant stress. As a result, maize plants exhibit limited efficiency in utilizing these reserves for grain filling prior to flowering (Kiniry et al., 1992). Instead, these reserves are more effectively used in post-flowering stages, such as during leaf senescence. In contrast, wheat demonstrates a three- to four-fold higher efficiency in the remobilization of assimilates stored before flowering for seed growth, in comparison to maize (Borrás et al., 2004). Initial stages of seed development, including cellularization and grain filling, are characterized by intense transcriptional regulation across various pathways. These pathways encompass primary metabolism, cell division, stress response mechanisms, and protein synthesis and degradation (Sprunck et al., 2005; She et al., 2011; Chen et al., 2017a). However, as seeds advance toward maturity, they develop a suite of crucial physiological characteristics that ensure successful seedling establishment once they are independent of the parent plant. These traits hinge on the ability to undergo complete desiccation without losing viability, a phenomenon referred to as desiccation tolerance (Righetti et al., 2015). Therefore, the focus of transcriptional regulation shifts from primary metabolism pathways to processes like chromatin condensation and nuclear size reduction (Baroux et al., 2007). At this stage, a mature grain is composed of starchy endosperm (accounting for 83–84% of dry weight), embryo (3%), aleurone layer (6.5%), and pericarp (7–8%), with only minor differences observed between wheat and maize (Barron et al., 2007; Sethi et al., 2021).

## 3 GWAS and QTL studies on seeds traits

QTL mapping is a statistical analysis to identify a region of DNA associated with a particular trait that exhibits variability within a population based on a genetic linkage map (Lander and Botstein, 1989). The mapping process requires the population generated between two genetically distinct parents with respect to the trait of interest (Myles and Wayne, 2008). In most of cases, a significant portion of the phenotypic variation within this region can be attributed to a small number of loci with substantial effects (Remington and Purugganan, 2003). The precisely mapping of QTL to a fine genetic position generally requires sufficient map resolution (Dinka et al., 2007).

With the emergence of high-throughput genotyping technologies, millions of markers can be identified at more affordable costs. GWAS has now become one of the most potent tools at the disposal of geneticists (Flint-Garcia et al., 2003). GWAS aims to identify associations between genotypes and traits of interest by leveraging phenotypic variations across diverse populations. These populations consist of unrelated individuals whose ancestry can be traced back to a common progenitor (Uffelmann et al., 2021). Consequently, the genetic distance between these individuals, influenced by numerous recombination events, leads to a faster decay in linkage disequilibrium, requiring extensive marker coverage to sufficiently capture the genome-wide genetic variations (Alqudah et al., 2020).

In recent decades, QTL mapping and GWAS have emerged as successful approaches, significantly advancing our understanding of gene functions in a variety of crops, including wheat, maize, and rice. It has been widely used to investigate a range of biological and physiological traits, as well as to uncover the genetic underpinnings of natural selection and population divergences within a species. There are numerous comprehensive reviews that delve into population design and statistical models used in breeding research (Bordes et al., 2014; Xiao et al., 2017; Shikha et al., 2021; Dwiningsih and Al-Kahtani, 2022; Gupta et al., 2022; Saini et al., 2022). Plant geneticists focus on a wide array of traits, ranging from disease resistance and grain yield to flowering time, biomass, and seed size, among others. Our review of literature from 2011 to the present reveals that disease resistance and grain yield are particularly prominent, accounting for over half of the studies published (773/1004 for wheat, 501/884 for maize). This emphasis highlights the critical importance of these two traits in agricultural research (Kaya and Akcura, 2014; Millet et al., 2019). (Bhatta et al., 2018; Qaseem et al., 2019). (Qaseem et al., 2019). (Muhammad et al., 2020).

Grain yield is a multifaceted trait, intricately linked to both the number of grains per unit area and the thousand-kernel weight (TKW). Furthermore, aspects like grain shape, spike architecture, plant height, and flag leaf size play a crucial role in influencing grain yield by affecting photosynthesis. In wheat research, a multitude of candidate loci have been identified, each associated with grain yield, size, and TKW. In the study conducted by Yu and colleagues, 768 bread wheat accessions were analyzed to pinpoint the genetic loci linked to grain size and weight. Notably, a principal locus related to grain length, designated as qGL-2D, exhibited the most robust association across multiple years and encompasses 125 annotated genes. Through transcriptome analysis, the researchers validated the gene *TraesCS2D02G414800*, which is responsible for encoding an oleosin—a protein crucial in seed maturation and germination—as a candidate gene within the qGL-2D locus (Yu et al., 2022). In a separate study conducted by Tekeu and team, three quantitative trait loci (QTLs) related to grain size were identified on chromosomes 1D, 2D, and 4A. This discovery was made through a GWAS that included 157 international wheat accessions and 71 Canadian wheat accessions. Notably, a candidate gene located on chromosome 2D, *TraesCS2D01G331100*, was identified as an ortholog of the D11 gene in rice. This gene, which encodes a cytochrome P450, displayed high expression levels in the developing embryo, particularly during embryogenesis and grain development in wheat, affecting both endosperm and pericarp tissues (Tekeu et al., 2021).

Although a vast array of candidate genes linked to grain yield have been discovered through GWAS and QTL mapping, the molecular cloning of these genes in wheat is still relatively unexplored (see **Tables 1**, **2**). Unlike wheat, maize is a diploid species, the research on seeds development has greatly benefited from the investigation of mutator-induced mutant collections such as *defective kernel* (*dek*) mutants with affected development in both embryo and endosperm (Neuffer and Sheridan, 1980; McCarty et al., 2005), *empty pericarp* (*emp*) mutants (Scanlon et al., 1994), *defective endosperm* (*de*) or *endosperm-specific* mutant (Consonni et al., 2022) and *embryo specific* (*emb*) mutants (Clark and Sheridan, 1991). Those mutant collections provide abundant material for the molecular genetic analysis on the mechanisms of embryo and endosperm development allowing maize to become the leading system for molecular cloning of functional genes in plants. For instance, numerous *dek* mutants, such as *Dek1* (encoding a membrane protein of the calpain gene superfamily) (Lid et al., 2002), *Dek2*, *Dek35*, *Dek36*, *Dek37*, *Dek39* (encode a pentatricopeptide repeat protein) (Qi et al., 2017; Wang et al., 2017; Chen et al., 2017b; Dai et al., 2018; Li et al., 2018), and Dek* (encoding AAA-ATPase controlling 60S ribosome export) were characterized. The associated pathways involve cell fate specification in the endosperm (*Dek1*), ribosome use efficiency (Dek*) and RNA editing (*Dek2, Dek35–39*). However, compared to the mutant collections, the number of genes identified still represents only a small fraction of the entirety of the collections.

**Table 1:** List of cloned genes associated with seed traits in wheat, this list only covers the genes identified through GWAS and QTL mapping.

Gene	Seed trait	Protein function	Reference
*TaGW2*	Grain size and weight	encoding a C5HC2-type E3 ubiquitin ligase	Su et al. (2011)
			Li et al. (2023)
			Zhang et al. (2018)
			Zhai et al. (2018)
*TaTEF-7A*	Kernel number per spike	a member of the transcript elongation factor gene family	Zheng et al. (2014)
*TaBT1*	Grain weight	transporter for the translocation of small molecules between the mitochondria and cytoplasm	Wang et al. (2019b)
*TaTPP-7A*	Grain weight	Trehalose 6-phosphate phosphatase	Liu et al. (2023)
*TaGS5–3A*	Grain weight	serine carboxypeptidase	Ma et al. (2016)
*TaDA1*	Grain weight	a ubiquitin receptor	Liu et al. (2020)
*TaCKX6-D1*	Grain weight	a cytokinin oxidase/dehydrogenase	Zhang et al. (2012)
*TaGASR7*	Grain weight	encoding a protein with a signal peptide that responsive to gibberellic acid	Zhang et al. (2016)
			Dong et al. (2014)
*TaAGP-S1–7A*	Grain weight	subunit of ADP-glucose pyrophosphorylase	Hou et al. (2017)
*TaAGP-L-1B*	Grain weight	subunit of ADP-glucose pyrophosphorylase	Hou et al. (2017)
*TaGW7*	Grain size and weight	encoding a TONNEAU1‐recruiting motif (TRM) protein	Wang et al. (2019a)
*TaGW6*	Grain weight	encoding a novel indole-3-acetic acid-glucose hydrolase	Hu et al. (2016)
*TaIAA21*	Grain size and weight	negative factor involved in Auxin signaling	Jia et al. (2021)
*TaHST1L*	Grain yield	encoding a NAD-dependent deacetylase involved in auxin signal	Zhao et al. (2023)

**Table 2:** Genomic positions of cloned genes associated with seed traits in wheat and maize through GWAS and QTL analysis.

Gene name	Gene ID	Physical position	Genome version
*TaDA1*	TraesCS2A01G007800	Chr2A:8319781 - 8326143	IWGSC CS RefSeq v2.0
*TaGW2*	TraesCS6A02G189300	Chr6A:240302888 - 240328295	IWGSC CS RefSeq v2.1
*TaEF*	TraesCS7A02G108900	Chr7A:69499125 - 69501354	IWGSC CS RefSeq v2.1
*TaBT1*	TraesCS6A02G175100	Chr6A:192285138 - 192288210	IWGSC CS RefSeq v2.1
*TaTPP-7A*	TraesCS7A03G0422300	Chr7A:138613354 - 138615932	IWGSC CS RefSeq v2.0
*TaGS5–3A*	TraesKAR3A01G0118380	Chr3A:188198450 - 188202484	Gramene release 68
*TaCKX6-D1*	TraesCS3D02G143300	Chr3D:85320853 - 85321389	IWGSC CS RefSeq v2.1
*TaGASR7-A*	TraesCS7A02G208100	Chr7A:175963414 - 175964402	IWGSC CS RefSeq v2.1
*TaAGP-S1–7A*	TraesCS7A02G287400	Chr7A:347014423 - 347022505	IWGSC CS RefSeq v2.1
*TaAGP-L-1B*	TraesCS1D03G0983500	Chr1D:482493243 - 482498148	IWGSC CS RefSeq v2.1
*TaGW7–1B*	TraesCS2B01G202300	Chr2B:190072020 - 190077273	IWGSC CS RefSeq v2.1
*TaGW7–1D*	TraesCS2D01G183400	Chr2D:130713880 - 130719328	IWGSC CS RefSeq v2.1
*TaGW6-B*	TraesCSU02G223800	ChrUnknown:249079212 - 249080545	IWGSC CS RefSeq v2.1
*TaIAA21-A*	TraesCS7A02G331100	Chr7A:488460835 - 488464693	IWGSC CS RefSeq v2.1
*TaIAA21-B*	TraesCS7B02G242800	Chr7B:455890694 - 455894566	IWGSC CS RefSeq v2.1
*TaIAA21-D*	TraesCS7D02G339300	Chr7D:435735509 - 435739416	IWGSC CS RefSeq v2.1
*TaHST1L*	TraesCS5A02G316400	Chr5A:403990309 - 403991592	IWGSC CS RefSeq v2.1
*ZmGS3*	GRMZM2G139878	Chr1:70729186 - 70731364	B73 RefGen_v3
*ZmYIGE1*	GRMZM2G008490	Chr1:50674670 - 50681478	B73 RefGen_v3
*ZmVPS29*	GRMZM2G068489	Chr4:224200238 - 224211317	B73 RefGen_v3
*ZmG6PE*	GRMZM2G039588	Chr2:4178376 - 4186062	B73 RefGen_v3
*ZmMSH7*	GRMZM2G110212	Chr3:9423562 - 9437621	B73 RefGen_v3
*ZmGW2*	GRMZM2G029690	Chr9:106080135 - 106087638	B73 RefGen_v3
*ZmGS5*	GRMZM2G123815	Chr3:61240172 - 61246469	B73 RefGen_v3
*ZmBZR1*	GRMZM6G287292	Chr3:5295292 - 5300354	B73 RefGen_v3
*ZmFAD2*	GRMZM2G056252	Chr5:203090777 - 203098676	B73 RefGen_v3
*ZmNAC78*	GRMZM2G406204	Chr1:4240471 - 4247496	B73 RefGen_v3
*ZmMADS26*	GRMZM2G044408	Chr5:210272259 - 210286975	B73 RefGen_v3
*ZmTPS9*	GRMZM2G312521	Chr4:174990784 - 174997304	B73 RefGen_v3
*ZmcrtRB1*	GRMZM2G152135	Chr10:136080567 - 136085686	B73 RefGen_v3
*ZmKW9*	GRMZM2G171994	Chr9:153757502 - 153762626	B73 RefGen_v3
*ZmBAM1d*	GRMZM2G043584	Chr1:30260694 - 30262694	B73 RefGen_v3

With the recent advancements of GWAS and QTL mapping, more natural genetic variations related to maize kernel traits had been identified (**Tables 2**, **3**). By comparing the genes list between wheat and maize, there are some genes orthologs exist in both wheat and maize that effected the grain weight and size, such as *Grain Size 5* (*GS5*) which encodes a serine-type carboxypeptidase. The overexpression of *GS5* in wheat and maize both exhibit a positive regulation of grain weight suggesting a similar pathway controlling the yield (Liu et al., 2015; Ma et al., 2016). Remarkably, a recent GWAS, which examined a Chinese mini-core collection comprising 262 accessions—encompassing over 70% of the genetic diversity in Chinese wheat—uncovered a notable candidate gene, trehalose-6-phosphate phosphatase (*TaTPP-7A*). This gene is significantly correlated with the TKW in wheat (Liu et al., 2023). Overexpression of *TaTPP-7A* in wheat induced a phenotype featuring higher TKW, longer grains and early maturity, while RNAi or gene-edited lines with silenced *TaTPP-7A* expression exhibited a reversed phenotype, including lower TKW, shorter grains, and late maturity. Further transcriptome analysis revealed that *TaTPP-7A* primarily boosts starch synthesis via the T6P-SnRK1 pathway and the sugar-ABA interaction. Intriguingly, the RAMOSA pathway, first proposed in maize decades ago, consists of three RAMOSA genes, including two transcription factors. The *RA1* gene encodes a Cys2-His2 zinc finger transcription factor, while *RA2* is responsible for a Lateral Organ Boundary (LOB) domain transcription factor. Meanwhile, *RA3* is identified as a gene encoding trehalose-6-phosphate phosphatase (TPP), which is expressed in the axillary inflorescence meristems (Wang et al., 2021). RA3 acts upstream of transcription factor RA1 to regulate inflorescence branching through modulation of treholose or T6P levels. In maize, two *TPP* genes, namely *RA3* and *TPP4*, have been characterized. Mutants of both *ra3* and *tpp4* demonstrated a decrease in crop yield, characterized by abnormal branching and irregular seed row patterns. Notably, the double mutant combining *ra3* and *tpp4* exhibited an even more pronounced phenotype (Satoh-Nagasawa et al., 2006; Claeys et al., 2019). Moreover, the overexpression of a rice *TPP* gene in the female reproductive tissues of maize significantly improved yield under drought conditions during the flowering stage (Nuccio et al., 2015). This, along with molecular evidence from mutants and transgenic lines in both wheat and maize, suggests a conserved pathway in cereal crops related to trehalose-6-phosphate (T6P), impacting grain size and yield.

**Table 3:** List of cloned genes associated with seed traits in maize, this list only covers the genes identified through GWAS and QTL mapping.

Gene	Seed trait	Protein function	Reference
*ZmGS3*	Grain size	a negative regulator of grain size with four functional differential domains	Li et al. (2010b)
*ZmYIGE1*	Grain weight, Ear length	unknown protein	Luo et al. (2022)
*ZmVPS29*	Grain shape and size	encoding a retromer complex component involved in auxin biosynthesis	Chen et al. (2020)
*ZmG6PE*	Grain weight	encoding a glucose-6-phosphate 1-epimerase	Zhang et al. (2023a)
*ZmMSH7*	Grain weight	a member of MutS-homologous (MSH) family of proteins involved in DNA mismatch repair	Jiang et al. (2024)
*ZmGW2*	Grain weight and size	encoding a RING-type protein with E3 ubiquitin ligase activity	Li et al. (2010a)
*ZmTB1*	Grain weight	encoding a member of the TCP family of transcriptional regulators	Clark et al. (2006)
*ZmGS5*	Grain weight	encoding a protein with serine-type carboxypeptidase activity	Liu et al. (2015)
*ZmBZR1*	Grain size	transcription factor	Zhang et al. (2020)
*ZmFAD2*	Grain oleic acid level	encoding a fatty acid desaturase	Beló et al. (2008)
*ZmNAC78*	Grain iron content	transcription factor	Yan et al. (2023)
*ZmMADS26*	Grain germination	a MADS-transcription factor 26	Ma et al. (2022)
*ZmTPS9*	Grain weight, Starch content	encoding a trehalose-6-phosphate synthase in the trehalose pathway	Hu et al. (2021)
*ZmcrtRB1*	Grain *β*-carotene content	encoding *β*-carotene hydroxylase 1	Yan et al. (2010); Yin et al. (2024)
*ZmKW9*	Grain weight	encoding a DYW motif pentatricopeptide repeat protein	Huang et al. (2020); Zhang et al. (2024)
*ZmBAM1d*	Grain weight	encoding a CLAVATA1 (CLV1)/BARELY ANY MERISTEM (BAM)-related receptor kinase-like protein	Yang et al. (2019)

In addition, increasing evidence has indicated the ubiquitin-proteasome pathway plays a conserved role in controlling the grain size through restricting the cell expansion in plants (Li and Li, 2016; Shi et al., 2019). *GW2* encodes a E3 ubiquitin ligase has been confirmed to be a negative regulators of grain size in rice (Song et al., 2007), maize (Li et al., 2010b) and wheat (Liu et al., 2020). Interestingly, a novel allele of *TaGW2* has been shown to increases grain weight but decrease grain number in wheat, suggesting additional roles of E3 ubiquitin ligase in controlling grain weight and size in wheat (Zhai et al., 2018).

Ortholog-based gene discovery is a valuable strategy, researchers often resort to identify orthologous genes from well-studied model plants. In the realm of cereal research, rice was widely used as a model species. In most cases, leveraging orthologs presents an effective avenue for gene discovery offering insights on conserved pathways and gene functions. However, it’s important to acknowledge that relying solely on ortholog-based gene discovery has its limitations. As the evolutionary distance between species can influence the accuracy of orthologs predictions, potentially resulting mis-annotations or missed genes and overlooking species-specific adaptations and unique genetic features (Woodhouse and Hufford, 2019).

Despite the identification of numerous candidate genes through GWAS that exhibit promising influences on grain traits such as ear length and height in maize, the precise roles of these genes remain elusive. For example, the gene *YIGE1*, implicated in sugar and auxin signaling pathways, was discovered via GWAS. CRISPR/Cas9 knockout mutants of *YIGE1* displayed reduced grain yield, accompanied by a decrease in female inflorescence meristem size and the number of kernels per row (Luo et al., 2022). Nevertheless, the specific function of the YIGE1 protein is yet to be fully characterized.

## 4 Understanding of seed development through transcriptomic, proteomic and metabolomic studies

### 4.1 Conserved regulatory principles in the grain filling stage during endosperm development

The endosperm, forming the bulk of the seed, acts as a reservoir for essential components, predominantly starch and proteins. Starch, as a primary energy source, accounts for 70% to 80% of the dry weight in cereal seeds, while proteins comprise about 10%. Owing to its crucial role, the endosperm has become a focal point in diverse omics research fields.

In wheat, a significant number of genes, precisely 46,487 out of the total 85,173, were found to be active during endosperm development. This figure is remarkably similar to that observed in maize, where 46,274 genes were expressed (Li et al., 2014; Pfeifer et al., 2014). The variation in endosperm cell differentiation between wheat and maize influences the preferential expression of genes in specific cell types and developmental stages. Nevertheless, within common cell types like the aleurone (AL) and starchy endosperm (SE), there is a notable uniformity in the expression of specific genes. Genes exclusive to AL are predominantly linked to lipid metabolism, carbohydrate metabolic processes, and amino acid biosynthesis. Conversely, genes specific to SE primarily focus on carbohydrate and saccharide metabolism (Woo et al., 2001; Gillies et al., 2012; Li et al., 2014; Pfeifer et al., 2014). Additionally, two significant studies centered on wheat grains utilized time-series metabolomic analysis in conjunction with proteomic techniques. These studies successfully uncovered the temporal and spatial distribution of proteins and metabolites within various cell types, providing a more nuanced understanding of their dynamics (Zhang et al., 2021; Zhang et al, 2023b). These studies identified distinct proteins and metabolites in four critical cell types: aleurone (AL), sub-aleurone (SA), starchy endosperm (SE), and endosperm transfer cells (ETC). Notably, during the essential 15-day period post-anthesis (DAA), a pivotal phase in grain development, carbohydrates such as sucrose, glucose, fructose, and myo-inositol showed significant accumulation in SE and ETC. Enzymes involved in carbohydrate interconversion, including beta-fructofuranosidase insoluble isoenzyme 2, 1,2-beta-Fructan 1*^F^*-fructosyltransferase, and sucrose synthase, exhibited a similar trend. This pattern aligns with the transcriptome and metabolome data (Zhang et al., 2023b). The findings from these studies suggest the presence of conserved regulatory principles within the cereal family.

While starch and protein are the primary constituents of cereal crops like maize and wheat, the distribution of these polymers during seed maturation exhibits spatial differences among various plant species. A striking example is evident in a proteomic profiling study of 16 cereal grains, which revealed significant variations in the gluten-enriched fractions of storage proteins between wheat and maize (Colgrave et al., 2015). Wheat varieties, along with barley and rye, predominantly accumulate high molecular weight glutenins, ranging from 35 to 180 kDa. Conversely, in maize, the gluten-enriched fractions chiefly consist of zeins, with molecular weights typically between 9 to 29 kDa. Such divergence in polymer distribution highlights the complexity of the seed maturation process across different cereal species.

### 4.2 Do wheat seeds employ a C4-type carbon concentrating mechanism like maize?

As a prototypical C4 plant, maize utilizes a specialized cell-type-specific expression pattern for photosynthesis, which allows for the spatial segregation of this process. Key enzymes such as phosphoenolpyruvate carboxylase (PEPC), malate dehydrogenase (MDH), pyruvate orthophosphate dikinase (PPDK), and carbonic anhydrase (CA) are predominantly expressed in the outer mesophyll cells. In contrast, enzymes like ribulose 1,5-bisphosphate carboxylase-oxygenase (Rubisco), dicarboxylic acid transporter (DCT), and NAD-dependent malic enzyme (NAD-ME) are primarily localized in the inner bundle sheath (BS) cells [(Weber and von Caemmerer, 2010; Gowik et al., 2011; Medeiros et al., 2021)]. C4 photosynthesis is designed to concentrate *CO*_2_ in the vicinity of the primary carboxylase, Rubisco, which can bind to *CO*_2_ or *O*_2_ in a reversible reaction. This mechanism significantly enhances photosynthetic efficiency by inhibiting photorespiration, a process that competes with photosynthesis.

In wheat, although the endosperm is incapable of photosynthesis, the surrounding green tissues, such as the pericarp and glumes, are crucial for supporting seed development via photosynthesis. The major contributors to grain filling are photosynthesis in the flag leaf and ear. Despite the pericarp displaying a bright green color during grain filling, it likely re-assimilates *CO*_2_ from respiration rather than direct photosynthesis due to limited stomata. In contrast, the glumes, which envelop the seed, possess a higher density of stomata and feature specialized Kranz cells around their vascular bundles. This morphology suggests a potential role for these cells in a form of C4 photosynthesis (Cochrane and Duffus, 1979).

In the field of omics research, particularly concerning the Poaceae family and the study of C3 and C4 plants, transcriptomic studies are of paramount importance. Recent RNA-Seq experiments spanning various wheat genotypes have yielded a wealth of data. During the mid-development stage of the seed, active photosynthesis in the pericarp is indicated by the expression of related genes, but this activity decreases as the seed matures (Rangan et al., 2017). Additionally, transcriptome analysis of wheat leaves and seeds revealed the expression of a C4-specific form of PEPC, suggesting the involvement of the C4 pathway in the pericarp, possibly to capture carbon released from endosperm respiration. Genes integral to C4 photosynthesis, including PEPC, MDH, NAD-ME, and PPDK, were identified, with all transcripts accumulating in the pericarp (Rangan et al., 2016). Consequently, it is hypothesized that C4 photosynthesis may play a role in grain filling in wheat, particularly via the pericarp.

Typically, assessments of mRNA abundance are common methodologies used to infer the proteotype. However, it has been clearly established that there is a tenuous correlation between mRNA levels and protein abundance (Petricka et al., 2012; Vogel and Marcotte, 2012). This is particularly true for metabolic enzymes, where protein functionality is complex and may vary due to amino acid substitutions affecting substrate and product relationships. Accurately characterizing different isoenzymes necessitates more in-depth carbon flux analyses and metabolomic studies, especially focusing on the pericarp. For instance, a previous experiment using radio-labeled ^14^ *CO*_2_ to trace carbon flux in wheat grains revealed that approximately 10% of the ^14^ *CO*_2_ was incorporated into the C4 acids malate and aspartate (Bort et al., 1995). Therefore, a more thorough analysis of carbon flux specifically from the pericarp would be particularly revealing.

To comprehensively understand the role of photosynthesis in grain filling within cereal crops, an integrated multi-omics approach that synthesizes insights from genomics, transcriptomics, proteomics, and metabolomics is essential. The current debate regarding the contributions of photosynthetic metabolism in wheat ears highlights the imperative for ongoing research. This research is necessary to unravel the complex regulatory mechanisms that govern these vital processes in cereal crops.

## 5 Conclusion

This review presents an in-depth exploration of the complex mechanisms involved in seed development in wheat and maize, with a particular focus on the molecular dimensions as revealed through genomics, transcriptomics, proteomics, and metabolomics. Despite considerable research on these individual species, there is a notable deficiency in comprehensive comparative studies between C3 and C4 plants within the Poaceae family. The conclusion emphasizes the essential need for a multidisciplinary approach to decode the complexities of photosynthetic contributions to grain filling in cereal crops. It draws attention to the ongoing debates within this field and underlines the critical importance of sustained research. Future research should aim to clarify the regulatory principles, particularly through an integrated lens of genomics, transcriptomics, proteomics and metabolomics.

## Author contributions

YJ: Conceptualization, Writing – original draft, Writing – review & editing. TH: Writing – original draft, Writing – review & editing. AS: Supervision, Writing – review & editing. LJ: Conceptualization, Supervision, Writing – review & editing.
